# The mediating effects of social support on the influencing relationship between grit and academic burnout of the nursing students

**DOI:** 10.1002/nop2.1241

**Published:** 2022-05-29

**Authors:** Hae‐Ok Kim, Insook Lee

**Affiliations:** ^1^ Department of Nursing Kyungnam University Changwon Korea; ^2^ Department of Nursing Changwon National University Changwon Korea

**Keywords:** burnout, grit, mediation analysis, nursing, social support, student

## Abstract

**Aim(s):**

This study aimed to examine the social supports' mediating effects on the relationship between grit and academic burnout in nursing students.

**Design:**

This was a descriptive study.

**Methods:**

This study was conducted in two nursing departments in Korea, utilizing a convenience sample of 158 nursing students. Data were analysed using hierarchical multiple regression analysis and bootstrapping to verify the mediation effect.

**Results:**

The grit's direct effect on academic burnout and indirect effect with social support on academic burnout were statistically significant. Social support mediated the relationship between grit and academic burnout. Social support can improve grit and reduce academic burnout. Social support programs for nursing students should be implemented to prevent/reduce academic burnout.

## 1 INTRODUCTION

Academic burnout is emerging as a global problem that bothers college students and has a significant impact on students' mental health and academic achievement (Rahmatpour et al., 2019; Salmela‐Aro et al., 2009), and there is a high risk of academic burnout. Korean nursing students obtain a nurse's licence after completing the curriculum, including clinical practice for more than 1,000 hr. Compared to other academic fields, nursing students have higher academic burnout while completing the standardized curriculum and theoretical and practical courses in intensive completion. Also, it has been reported that there are many complicated parts due to various clinical field practice, so they feel a heavier academic burden than college students in other majors and experience much stress due to this (Cho et al., 2016; Deary et al., 2003; Jung & Jeong, 2018). It is said that students' academic burnout is experiencing the same level of burnout as office workers, and they are experiencing intense burnout (Lee & An, 2014). Nursing students' academic burnout is a severe problem for nursing students who have to complete a large amount of learning and practice; it is necessary to identify factors to prevent and reduce their academic burnout and void academic burnout.

Academic burnout is a psychological condition such as emotional burnout, cynicism, and academic inefficiencies caused by the inability to effectively manage academic pressure, and not meet academic requirements (Schaufeli et al., 2002). For this reason, there is a characteristic of low motivation and an increased feeling of failure (Salmela‐Aro et al., 2009). In other words, academic burnout is a psychological state caused by academic stress and academic burden that has been received over a long period and is manifested by emotional exhaustion, a cynical attitude and a sense of distance towards study, and helplessness (Park et al., 2010). Failure to adapt to the nursing department's course due to an academic burnout may lead to discontinuation problems or drop out of school (Lee & Park, 2018). There is a need to improve school adaptability by reducing academic burnout's negative impact and making the learning experience positive (Deary et al., 2003).

As a result of previous studies, it was reported that the variables affecting college students' academic burnout were related to personal personality aspects and interpersonal relations such as social support (Park et al., 2010); however, there is insufficient understanding of whether it is triggered (Lee & An, 2014). Because studies on academic burnout of nursing students are still incomplete, a systematic intervention will be possible so that students do not experience difficulties in academic performance due to academic burnout by examining the academic burnout of nursing students through this study and identifying the factors that influence their academic burnout.

Grit is a passion and perseverance for long‐term goals (Duckworth et al., 2007), and is known for maintaining high well‐being despite adversity and failure in the long‐term goals with an enduring passion for specific interests and persevering in pursuing long‐term goals (Duckworth et al., 2007; Duckworth & Quinn, 2009; Lim, 2017a). Grits have been found to play a positive role in individuals' psychological health and positively affect students and the general public (Salles et al., 2014; Vainio & Daukantaitė, 2016). Therefore, to reduce academic burnout, which is a negative experience that students feel in the nursing colleges' curriculum, it is necessary to understand each learner's positive psychological attribute and grit; and increase it.

Social support is known to act as a social and psychological protective factor, reducing the adverse effects of stress on the individual's physical and mental health (Glanz et al., 2008). Social support has been reported to reduce students' academic burnout (An et al., 2017; Noh, 2017) and act as a potential effect variable in the relationship between students' grit and academic achievement (Clark et al., 2020). Therefore, it can be considered that social support acts as a factor that helps to reduce academic burnout and can mediate the relationship between grit and academic burnout.

As summarizing the results of previous studies, it can be predicted that nursing students' perceived social support strengthens grit to make students perceive less academic burnout; and buffers grit's effect on academic burnout, thereby reducing academic burnout. Until now, studies dealing with college students' academic burnout have been limited to the exploratory level of finding the variables that affect academic burnout and analysing their influence. However, it did not analyse how each variable affected nursing students' academic burnout. In particular, it was challenging to find a study that verified the mediating effect among variables related to academic burnout.

The mediating effect examines the independent variable's influence on the dependent variable through the mediating variable. If this study understands the social supports' mediating effect, it will be possible to find an effective way to reduce academic burnout.

### 1.1 Research objectives

This study aimed to examine the relationships between grit, social support, and academic burnout in nursing students; and identify the social supports' mediating effects in the relationship between grit and academic burnout. Social support can act as a mechanism for generating influence in grit's impact on academic burnout. Therefore, this study will provide the basis for devising practical and useful intervention strategies to prevent/reduce nursing students' academic burnout. The specific research objectives are as follows: (i) identify nursing students' grit, social support, and academic burnout; (ii) understand the relationship between grit, social support, and academic burnout; (iii) examine the effect of grit on academic burnout; and (iv) verify the effect of nursing students' grit on academic burnout through social support.

## 2 METHODS

### 2.1 Design

This study used a descriptive survey design to test social supports' mediation effect.

### 2.2 Participants

Participants were conveniently sampled of junior and senior nursing students from two colleges in C city, South Korea. Among the 160 nursing students who agreed to participate, 158 surveys were collected (response rate: 98.8%). Using the G*Power 3.1.9.2 program, the number of research participants calculated with a significance level (α) of .05; power (1‐β) of .95; effect size (f^2^) of .15; and five predictors. The resulting sample size was 138. Data were collected from 160 participants considering the dropout rate of 15%.

### 2.3 Measures

#### 2.3.1 Grit

Duckworth (2016) grit scale was used with a 7‐point Likert scale of 10 questions to measure grit; nursing students' psychological attributes. This scale consists of five‐items of interest, which measure the degree of interest consistently, and five‐items of the persistence of effort measure the degree of the continuous effort to achieve long‐term goals. Cronbach's α at the time of development was .85 (Duckworth et al., 2007), and Cronbach's α in Lim's (2017a) research was .70 for interest retention, .98 for the endurance of effort, and .81 for the whole. Cronbach's α in this study was .71.

#### 2.3.2 Social support

Zimet et al.'s (1988) multidimensional scale of perceived social support (MSPSS) was used with a 7‐point Likert scale of 12‐item to measure the social support. MSPSS assessed the social supports' three dimensions: family, friends, and significant others. In this study, significant others were revised by dedicated professors or nursing professors. Cronbach's α in Zimet et al.'s (1988) research was .91. Cronbach's α in this study was .90.

#### 2.3.3 Academic burnout

Academic burnout scaled the Maslach burnout inventory‐student survey (MBI‐SS) developed by Schaufeli et al. (2002) as a student burnout scale and adapted and validated by Shin et al. (2011). This scale is a 5‐point Likert scale with a total of 15 questions: five‐items for emotional exhaustion, four‐items for cynicism, and six‐items for reduced academic efficacy. Cronbach's α in Schaufeli et al.'s (2002) research was .87; Cronbach's α in Shin et al.'s (2011) research was .82–.86. Cronbach's α in this study was .85.

## 3 DATA COLLECTION

A survey was conducted for 2‐weeks from December 9 to December 20, 2019. After IRB approval, data collection started. Questionnaires were distributed after receiving written informed consent to the subjects voluntarily participating in this study. Subjects who agreed to participate in this study received a gift worth KRW 3,000, but there were no factors that could interfere with the survey response.

## 4 ETHICAL CONSIDERATIONS

This study was conducted after Kyungnam University Institutional Review Board (IRB) approval for exemption from review (approved no. 1040460‐A‐2019‐059). For participants' ethical considerations, the researcher explained the research objectives and informed the participants that results might be used only for research purposes. Participants provided written informed consent before completing the study questionnaire.

## 5 DATA ANALYSIS

Data were analysed using SPSS/WIN 25.0 and Hayes' process macro ver. 3.4.1:

- Participants' general characteristics, grit, social support, and academic burnout were analysed using descriptive statistics.
- Independent *t*‐tests and one‐way analysis of variance (ANOVA) were used to identify the differences in grit, social support, and academic burnout according to the general characteristics.
- Correlations between grit, social support, and academic burnout were analysed using Pearson's correlation coefficient.
- Hierarchical multiple regression analyses were conducted to identify the social supports' mediating effects in the relationship between grit and academic burnout, and then process macro model 4 proposed by Hayes (2013), mediation analysis based on regression analysis was performed. The multicollinearity between independent variables checked the autocorrelation of error terms (Durbin‐Watson). Normality testing of the variables (Shapiro–Wilk test) and equal variance (Breusch‐Pagan test) were checked. The significance of the social supports' mediating effects was verified using the bootstrapping method through the 95% confidence interval and 5,000 bootstrap samples. The mediating effect size was estimated by checking the proportion of mediation (PM).

## 6 RESULTS

### 6.1 General characteristics

Participants were mostly female students (81.6%), with a mean (SD) age of 22.11 ± 1.32. The percentages of satisfaction with nursing major were 78.8%. The credits were 67.1% for the high, 23.4% for the medium, and 9.5% for the lower. The reasons for choosing a nursing major were 47.8% because of the employment rate, 15.3% because they want a professional job, and 36.9% such as a recommendation from others (Table 1).

**TABLE 1:** General characteristics and grit, social support, and academic burnout of subjects (*N* = 158)^a^

Variables	Categories	*N* (%)/mean ± SD	Skewness	Kurtosis
Gender	Male	29 (18.4)		
	Female	129 (81.6)		
Age (yr)	Range:	22.11 ± 1.32		
	≤21	53 (33.5)		
	≥22	105 (66.5)		
Grade	Junior	81 (51.3)		
	Senior	77 (48.7)		
Personality	Introverted	95 (60.1)		
	Extroverted	63 (39.9)		
Interpersonal relations	Good	89 (56.3)		
	Below average	69 (43.7)		
Satisfaction with major	Dissatisfaction	33 (21.2)		
	Satisfaction	123 (78.8)		
GPA	High (≥4.0)	106 (67.1)		
	Medium (≥3.0 ~ <4.0)	37 (23.4)		
	Low (<3.0)	15 (9.5)		
Reason for selection of nursing	Employment rate	75 (47.8)		
	Nursing profession	24 (15.3)		
	Others' recommendation, etc.	58 (36.9)		
Grit	Range: 10 ~ 70	40.76 ± 6.56	0.73	2.10
Social support	Range: 12 ~ 84	60.05 ± 11.08	−0.45	1.10
Academic burnout	Range: 15 ~ 75	43.55 ± 8.57	−0.29	1.03

Abbreviation: GPA, grade point average.

^a^ Missing data excluded.

### 6.2 Grit, social support, and academic burnout

As the descriptive statistics for grit, social support, and academic burnout (Table 1), the average grit score was 40.76 (*SD* = 6.56), and the average academic burnout score was 43.55 (*SD* = 8.57). The average social support score was 60.05 (*SD* = 11.08).

### 6.3 Differences in grit, social support, and academic burnout according to general characteristics

As the analysis of differences in grit, social support, and academic burnout according to general characteristics (Table 2), there were significant differences in grit by gender (t = 2.15, *p* = .033), satisfaction with nursing major (t = −2.01, *p* = .047). Grit was higher for subjects with a male than females when they were satisfied with their major. Social support had statistically significant differences personality (t = −2.06, *p* = .041), interpersonal relationship (t = 3.13, *p* = .002), and satisfaction with major (t = −2.36, *p* = .023). Social support was higher for subjects with an extroverted personality, smooth interpersonal relationships, and satisfaction with major. Academic burnout significantly differed according to grade (t = 3.47, *p* = .001), personality (t = 3.35, *p* = .001), satisfaction with major (t = 6.83, *p* < .001). Participants had higher academic burnout when they were junior; and had introverted personalities, dissatisfaction with major compared to satisfaction.

**TABLE 2:** Grit, social support, and academic burnout by general characteristics of subjects (*N* = 158)^a^

Variables	Categories	Grit		SS		AB	
		Mean ± SD	t/F (*p*)	Mean ± SD	t/F (*p*)	Mean ± SD	t/F (*p*)
Gender	Male	43.10 ± 6.53	2.15 (.033)	61.17 ± 15.64	0.46 (.651)	41.55 ± 10.72	−1.16 (.253)
	Female	40.23 ± 6.48		59.78 ± 9.81		44.01 ± 7.98	
Age(yr)	≤21	40.00 ± 6.62	−1.03 (.303)	58.69 ± 10.00	−1.08 (.282)	45.44 ± 7.31	1.96 (.052)
	≥22	41.14 ± 6.53		60.72 ± 11.57		42.62 ± 9.01	
Grade	Junior	40.40 ± 6.30	−0.72 (.476)	59.99 ± 10.10	−0.07 (.947)	45.80 ± 8.09	3.47 (.001)
	Senior	41.14 ± 6.84		60.11 ± 12.09		41.22 ± 8.47	
Personality	Introverted	40.23 ± 6.66	−1.24 (.215)	58.56 ± 10.19	−2.06 (.041)	45.37 ± 8.29	3.35 (.001)
	Extroverted	41.56 ± 6.37		62.24 ± 12.02		40.84 ± 8.30	
Interpersonal relations	Good	41.15 ± 7.06	0.84 (.402)	62.42 ± 10.33	3.13 (.002)	42.63 ± 8.67	−1.54 (.125)
	Below average	40.26 ± 5.87		56.97 ± 11.33		44.74 ± 8.34	
Satisfaction with major	Dissatisfaction	38.79 ± 4.82	−2.01 (.047)	55.31 ± 13.55	−2.36 (.023)	51.39 ± 7.09	6.83 (<.001)
	Satisfaction	41.35 ± 6.89		61.36 ± 10.10		41.31 ± 7.63	
GPA	High (≥4.0)	41.27 ± 6.85	1.56 (.214)	61.10 ± 10.13	1.51 (.225)	42.66 ± 8.78	2.75 (.067)
	Medium (3.0 ~ 3.9)	40.32 ± 5.87		58.08 ± 12.01		44.32 ± 8.47	
	Low (<3.0)	38.20 ± 5.72		57.29 ± 14.62		47.93 ± 5.71	
Reason for selection of nursing	Employment rate	40.08 ± 6.48	2.22 (.112)	59.31 ± 10.01	0.40 (.669)	45.08 ± 8.15	2.51 (.085)
	Nursing profession	43.29 ± 7.85		59.71 ± 10.96		43.00 ± 10.22	
	Recommendation,etc.	40.64 ± 5.97		61.05 ± 12.57		41.77 ± 8.20	

Abbreviations: AB, academic burden; GPA, grade point average; SS, social support.

^a^ Missing data excluded.

### 6.4 Correlation between grit, social support, and academic burnout

As the results of the correlation analyses (Table 3), academic burnout negatively correlated with social support (*r* = −.337, *p* < .001) and grit (*r* = −.400, *p* < .001); social support positively correlated with grit (*r* = .208, *p* < .001).

**TABLE 3:** Correlation among grit, social support, and academic burnout (*N* = 158)

Variables	Grit	SS	AB
	*r* (*p*)	*r* (*p*)	*r* (*p*)
1. Grit	1		
2. SS	.208 (.009)	1	
3. AB	−.400 (<.001)	−.337 (<.001)	1

Abbreviations: AB, academic burden; SS, social support.

### 6.5 Social supports' mediating effect

Before verifying the social support's mediating effects, this study checked the multicollinearity among variables, the error terms' autocorrelation (Durbin‐Watson), variables' normality testing (Shapiro–Wilk test), and equal variance (Breusch‐Pagan test). The residual limit was higher than 0.1, and the variance inflation factor's value was lower than 10. The Durbin‐Watson test indicated d = 2.166, which met the independence condition. The normality (Shapiro–Wilk test) was confirmed as *p* = .357 (*p* > .05); equal variance (Breusch‐Pagan's test) was confirmed as *p* = .550 (*p* > .05).

The hierarchical regression was used to confirm the social supports' mediating effects in the process of grit influencing academic burnout (Table 4). The result of the first step indicated that the independent variable (dummy variable: grad, personality, satisfaction with major) significantly affected academic burnout (senior; B = −4.39, *p* < .001, extroverted personality; B = −4.05, *p* = .001, satisfaction with major; B = −9.62, *p* < .001). The result of the second step indicated that the independent variable (grit) significantly affected the academic burnout (B = −0.42, *p* < .001), and the result of the third step indicated that the mediator variable (social support) significantly affected the academic burnout (B = −0.13, *p* = .011).

**TABLE 4:** Factors influencing academic burnout (*N* = 158)

Variables	Model I		Model II		Model III	
	B	t (*p*)	B	t (*p*)	B	t (*p*)
Constants	54.85	39.37 (<.001)	71.05	20.67 (<.001)	76.41	19.25 (<.001)
Grade: senior (0 = junior)	−4.39	−3.89 (<.001)	−4.08	−3.90 (<.001)	−4.11	−4.01 (<.001)
Personality: extroverted (0 = Introverted)	−4.05	−3.51 (.001)	−3.69	−3.44 (.001)	−3.31	−3.12 (.002)
Satisfaction with major (0 = Not Satisfied)	−9.62	−6.90 (<.001)	−8.76	−6.72 (<.001)	−8.12	−6.24 (<.001)
Grit			−0.42	−5.08 (<.001)	−0.38	−4.66 (<.001)
SS					−0.13	−2.57 (.011)
F (*p*)	27.07 (<.001)		30.12 (<.001)		26.32 (<.001)	
Adj. *R* ^2^	.338		.432		.453	
*R* ^2^ change			.096		.024	

Abbreviation: SS, social support.

**FIGURE 1:**
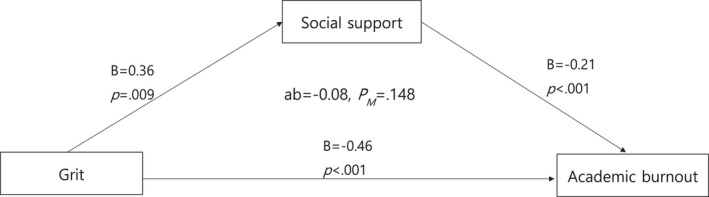
Mediating effect of social support on the relationship between grit and academic burnout

When mediation analysis was performed using SPSS process macro model 4 and bootstrapping to verify the significance of social supports' mediating effects (Table 5, Figure 1), grit was found to have a significant effect of −0.46 academic burnout within the 95% confidence interval. Within the 95% confidence interval, it was found that grit had a significant effect on social support by 0.36 and social support by −0.21 on academic burnout. Therefore, it can be seen that grit has a significant effect on academic burnout as much as −0.08 through social support. PM, the ratio of the indirect effect to the total effect, was 0.148, which means that 14.8% of grit's total impact on academic burnout is due to indirect effects through social support. This study executed bootstrapping using process macro model 4 to confirm the significance of social supports' mediating effects, confirming the relationship between grit and academic burnout. It can be seen that grit negatively affects academic burnout through social support.

**TABLE 5:** Results of mediating effect of social support by bootstrapping (*N* = 158)

Effect	Variables	B	SE	t	*p*	95% CI		*P*_*M*_
						LLCL	ULCL	
Direct effect	Grit → AB (c')	−0.46	0.10	−4.78	<.001	−0.656	−0.272	0.148
Indirect effect	Grit → SS (a)	0.36	0.14	2.64	.009	0.090	0.628	
Indirect effect	SS → AB (b)	−0.21	0.06	−3.65	<.001	−0.317	−0.094	
Indirect effect	Grit → SS → AB (ab)	−0.08	0.03			−0.144	−0.019	
Total effect	(c' + ab)	−0.54	0.10	−5.44	<.001	−0.733	−0.343	

*Note*: 5,000 samples re‐extracted for bootstrap.

Abbreviations: AB, academic burden; CI, confidential interval; LLCI, the lower limit of B in 95% confidential interval; P_M_, proportion mediated, ratio of the indirect effect to the total effect; SS, social support; ULCI, the upper limit of B in 95% confidential interval.

## 7 DISCUSSION

This study was confirmed the social supports' mediating effects in nursing students' grit and academic burnout. Based on these results, we tried to develop specific measures to prevent their academic burnout.

As a result, it was found that males had higher grit than females, and those who were satisfied with their major were more than dissatisfied. According to Hodge et al. (2018), there was no gender difference in grit among college students, which was different from this result. Meanwhile, grit tends to be higher in male students than female students but does not show inconsistent results (Jin & Kim, 2017; Jung & Jeong, 2018; Kang, 2018). Therefore, it is necessary to confirm grit's gender difference through repeated studies later. Also, grit was found to be high in the case of satisfaction with major, and this result was also found in the studies of Stoffel and Cain (2018), Jung and Jeong (2018), and Jin and Kim (2017) that when the satisfaction with major was high, grit increased. Therefore, it is necessary to develop grit so that nursing students can continue to pay attention to their majors and continuously realize their long‐term career development goals as professional nurses.

As for social support, extroverted personality was higher than introverted personality, a good interpersonal relationship was higher than average, and satisfaction with major was higher than dissatisfaction. In Noh's (2017) study, satisfaction with major showed high social support, which supported this result.

Academic burnout was 43.55 (mean score 2.90), although the scale was different, which was higher than that the burnout of nursing students in clinical practice was 2.54 points (Noh, 2017), and the academic burnout of nursing students in China was 2.77 points (Wang et al., 2019), and academic burnout of university students in the health and medical field of Iran was 2.53 points (Rahmatpour et al., 2019). However, it was similar to the nursing students' academic burnout 2.81 ~ 3.01 points (An et al., 2017; Cho & Kang, 2018), 2.95 points of nursing students in clinical practice (Lim, 2017), and healthcare majored students' academic burnout 2.97 points (Jang et al., 2019). Nevertheless, it was lower than the 4.00 points of academic burnout of nursing and oriental medicine students in Korea (Kim et al., 2019). It was shown that Korean college students' academic burnout is higher than that of other countries, so they need attention to pay.

In this study, academic burnout was higher in the junior than in the senior; this result was consistent with An et al.'s (2017) result. The group with the highest probability of experiencing academic burnout was in the freshman and senior grades, and there was a significant difference from this study as a result of male students (Aguayo et al., 2019). Moreover, the senior nursing student showed higher academic burnout than the first and third (Cho & Kang, 2018). However, grades did not significantly affect nursing students' academic burnout (Jang et al., 2019). As a result of Cho and Kang (2018), it was reported that nursing students had high burnout when they had clinical practice experience.

In junior nursing students in Korea, the department of nursing offers theoretical lessons from 8–10 weeks in the form of intensive completion from the 3rd year, and the remaining 6–7 weeks are operated by clinical field practice while other major students have 15 weeks of instruction in one semester. Also, it is thought that academic burnout in the junior nursing student is high due to changing the practice place before adapting because the clinical practice place changes every 1–2 weeks, and have to adapt to a different academic system from their first and second grades. In senior graders, they believe that they obtain their self‐management skills and tips for learning, which arise based on their junior experiences. Also, in the senior graders, the nursing department's clinical practice and academic curriculum are almost finished, and the employment has been decided, so it can be inferred that the academic burnout was low. Based on this result, it was found that there is a need to prepare preventive interventions at the university to prevent early academic burnout of junior nursing students, and individual nursing students need to manage their academic burnout through physical fitness and advance learning in the nursing major. If nursing students develop their way of managing academic burnout from nursing students, they will well manage burnout and stress even if they become nurses. Since academic burnout for the grade differences is inconsistent, nursing educators need to conduct repeated studies to understand nursing students' academic burnout by grade.

In this study, it was found that female students had high academic burnout, but there was no significance in gender. That is, nursing students experience academic burnout regardless of gender. These results are consistent with Kim et al. (2019), a study of university students in healthcare, and Lee and Jeon (2015) targeting medical students. Meanwhile, the results of this study were different from Cho and Kang (2018), which indicated that female students had high burnout, and from Aguayo et al. (2019), male students in the 4th grade of nursing with low personal achievement were at risk of academic burnout, and gender, major, and grade were positively related to academic burnout. These differences can be considered to be due to socio‐cultural differences, and it is necessary to confirm the gender differences in academic burnout through repeated studies.

In terms of academic burnout, the introverted personality was higher than the extroverted personality. A longitudinal study of nursing students found that students with low emotional stability were more likely to exhaust during academic burnout (Deary et al., 2003). The higher the integrity, extroversion, and affinity, personality factor, the less academic cynicism, and academic incompetence (Park et al., 2010). The positive psychological capital has a significant negative effect on academic burnout (Zhuang et al., 2020). Also, academic burnout was found to be high in cases of dissatisfaction with the major, and this result is similar to those of study that shows that students who are dissatisfied with their university life and who are dissatisfied with their nursing major have high academic burnout (Cho & Kang, 2018). These results can be inferred that students with introverted personalities or dissatisfaction with their major may be vulnerable to academic burnout because people who are not emotionally stable are psychologically unstable and are prone to psychological stress (Maslach et al., 2001). Therefore, it is necessary to provide psychological stability through social support by paying more attention to students who may be vulnerable to academic burnout.

In this study, there was no significant difference in academic burnout according to interpersonal relationships; however, due to previous studies, the absence of a stress coping method increases academic burnout (Cho & Kang, 2018). Therefore, to manage students' academic burnout, it is necessary to effectively control/manage academic burnout through efforts to strengthen interpersonal competence, which is a social support aspect.

As a result of examining the relationship among the subjects' grit, social support, and academic burnout, academic burnout negatively correlated with social support; however, social support was found to correlate with grit significantly. Factors influencing academic burnout were grade, personality, satisfaction with major, grit, and social support. An et al.'s (2017) research showed that nursing students' social support negatively correlated with academic burnout. Although the subjects were different, Kim and Jang's (2020) result showed that college students' grit had a significant negative correlation with academic burnout. Eskreis‐Winkler et al.'s (2014) research showed a positive correlation between grit and social support. According to Jang et al.'s (2019) research results, it was found that the academic burnout of nursing students was significantly affected by gender, academic enthusiasm, and sharing of learning materials, and they discussed that not only individual academic enthusiasm but also interpersonal aspects such as the degree of sharing of learning materials contribute to the reduction of overall academic burnout. This study found that grades, personality, and satisfaction with major significantly affected academic burnout. In contrast, gender and interpersonal relationships had no influence, so there was a difference. Therefore, it is necessary to confirm the factors influencing nursing students' academic burnout through repeated studies.

As a result of the mediation analysis, it was found that grit had a significant effect directly on academic burnout, and grit negatively affects academic burnout through social support. It was also confirmed that 14.8% of grit's total effects on academic burnout were due to indirect effects through social support. It can be seen that social support is an essential variable in reducing academic burnout among nursing students based on these results. Social support played a partial mediating role in reducing nursing students' practical stress and burnout (Noh, 2017). The results show that the interpersonal factor, a concept similar to social support, affects university students' reduction of academic burnout in the healthcare field (Jang et al., 2019). Jang et al. (2019) argued that if interpersonal relations, which can be called social aspects for nursing students, become better, academic burnout can be reduced, and to reduce academic burnout, it is necessary to consider not only personal aspects but also social aspects, interpersonal relationships (Jang et al., 2019). Lee and An (2014) discussed that in addition to demand, control, effort, and reward, social support and extraversion could reduce academic burnout. Therefore, nursing educators need to be interested in nursing students' academic burnout; and strengthen social support, including interpersonal relations, to reduce student burnout.

As a result of a longitudinal study conducted on medical students (Jumat et al., 2020), grit was found to be a strong predictor of burnout, and students with less grit were found to be vulnerable to burnout, which can be understood that the context of this research result is the same. On the other hand, some scholars mention that grit's effect on the relationship between grit and performance is somewhat exaggerated, and this problem is said to be because the measurement of grit is not consistent with the conceptual definition. Grit is defined as a combination of perseverance and passion, but the actual measurement of grit focuses on perseverance, and it is said that it is not adequately measured for passion (Jachimowicz et al., 2018; Lim, 2017b).

In the meta‐analysis of grit (Jachimowicz et al., 2018), it was confirmed that passion is a critical component of grit, and said that patience without passion is not grit; it is just hard work. Therefore, by integrating passion in the conceptualization and measurement of grit, it is said that by appropriately measuring both patience and passion, grit's true predictive power can be found (Jachimowicz et al., 2018). Credé et al.'s (2017) meta‐analysis and many other studies also showed a difference between the persistence of effort, which is the attribute of grit, and the persistence of interest, which is passion. In other words, it was found that passion had less explanatory power for various adaptive outcomes, such as subjective well‐being, self‐efficacy, and academic enthusiasm, compared to the steady effort (Datu et al., 2016; Jordan et al., 2015; Wolters & Hussain, 2015).

Duckworth (2016) argued that the persistence of interest, which is a passion, emphasizes how consistently individuals are immersed in their goals and interests. However, the existing grit scale, which simply focuses on the characteristics of maintaining and sustaining goals and interests, has limitations in that it cannot reliably and reasonably measure cultural differences (Datu et al., 2016; Datu et al., 2017) and passion as a reason for continuing action to achieve goals. Therefore, a deeper understanding of grit's conceptual structure and properties is needed, and the development and validation of the grit scale supplemented to reflect the conceptual properties of grit well are necessary.

The grit is almost unchanged and consistent after adulthood (Vazsonyi et al., 2019). Among the two aspects of grit, patience positively predicts academic achievement, hopes, positive psychological states, and family relationships nurturing grit (Christopoulou et al., 2018). Therefore, there is a need to provide positive experiences of various activities so that nursing students can develop grit before they become fixed; and strengthen social support through professors and friends at university. The intervention program needs to constitute a program element that develops the strength to endure the psychological shock caused by stress considering the risk factors affecting academic burnout and the protective factors buffering academic burnout symptoms (Lee & An, 2014). Therefore, it is necessary to reinforce the grit that nursing students can maintain their interest in their majors and continue their efforts to become professional nurses, and develop a compensation system appropriate for academic performance. Furthermore, more active strategies to promote social support, such as academic mentoring and senior and junior mentoring, and periodic personal counselling on academic studies, will be needed.

This study is meaningful as a study that empirically confirmed that it is possible to reduce academic burnout through social support and increase nursing students' grit through social support by identifying the social supports' mediating effect. Students experienced academic burnout through the nursing curriculum; providing a familiar and comfortable learning environment to practice clinical nursing and learn nursing curriculum can enhance the grit and prevent/reduce academic burnout. In particular, mentoring and coaching programs in the nursing curriculum may effectively support nursing students, and can be a helpful program for the gritty students.

## 8 CONCLUSION

This result shows that social support mediates the relationship between grit and academic burnout in nursing students. This finding has a tremendous impact on improving nursing academic performance, especially in how it relates to academic burnout, and they also show how academic burnout can be decreased. This result can be used as primary data to develop educational programs to improve nursing students' academic burnout. Therefore, it is necessary to develop and apply intervention strategies to enhance social support, thereby increasing grit and preventing/reducing nursing students' academic burnout. A social support program for the nursing students may enormously contribute to enhancing the grit and preventing/reducing academic burnout by mentoring and coaching them with adequate informational and emotional support.

However, it is difficult to generalize these results to all nursing students in that this study was conducted only for nursing students in a region. In future research, it is necessary to increase the possibility of generalization of research results through multi‐center‐multi‐regional research and conduct qualitative research to understand the nature of nursing students' academic burnout.

## 9 RELEVANCE FOR CLINICAL PRACTICE

This study is meaningful because it can contribute to developing a practical nursing curriculum to reduce nursing students' academic burnout, and prepare an efficient curriculum plan. It is also meaningful to confirm social supports' role by analysing whether social support plays a mediating role in the relationship between grit and academic burnout. This study was shown a necessary further study that develops social support supporting programs for nursing students and tests the effect of that.

## AUTHOR CONTRIBUTIONS

*Conceptualization and Study design:* Insook Lee. *Data collection:* Hae‐Ok Kim and Insook Lee. *Data analysis:* Insook Lee. *Visualization:* Insook Lee. *Manuscript writing: original draft or/and review & editing:* Hae‐Ok Kim and Insook Lee.

## CONFLICT OF INTEREST

“No conflict of interest has been declared by the author(s).”

## CONSENT FOR PUBLICATION

All participants consented and signed informed consent regarding publishing their anonymous recorded data, and informed consent was obtained from all individual participants for whom identifying information (gender, age, etc.) was included in this article.

## Data Availability

The datasets generated and/or analysed during the current study are not publicly available due to restrictions applied to the availability of these data, which were used under licence for the current study. Data are, however, available from the corresponding author upon reasonable request.
